# Functional organization and dynamics of the cell nucleus

**DOI:** 10.3389/fpls.2014.00378

**Published:** 2014-08-12

**Authors:** Tongtong Guo, Yuda Fang

**Affiliations:** National key Laboratory of Plant Molecular Genetics, Shanghai Institute of Plant Physiology and Ecology, Shanghai Institutes for Biological Sciences, Chinese Academy of SciencesShanghai, China

**Keywords:** lamina, LINC complex, NPC, genome architecture and expression, nuclear bodies

## Abstract

The eukaryotic cell nucleus enclosed within the nuclear envelope harbors organized chromatin territories and various nuclear bodies as sub-nuclear compartments. This higher-order nuclear organization provides a unique environment to regulate the genome during replication, transcription, maintenance, and other processes. In this review, we focus on the plant four-dimensional nuclear organization, its dynamics and function in response to signals during development or stress.

## INTRODUCTION

In recent years, a growing number of studies utilizing advanced imaging and Chromosome Conformation Capture (3C)-based techniques have further revealed the hierarchical organization of the chromosomes inside the cell nucleus, and suggested that the eukaryotic genome is territorially organized and the genes may be regulated by chromatin looping and interchromosomal contacts (Deng and Blobel, 2014). It seems that each gene is surrounded by a number of potential regulatory elements in the very crowded nucleus, raising a major question: how do cells ensure that genes respond to the right elements and avoid mis-regulation (Dekker et al., 2013b). Spatial-temporal organization of cell nucleus enables to achieve the required subtle and specific regulation in the crowded nucleus. For more detailed view of the nuclear biology, the readers are referred to the recent reviews, “The Dynamic Nucleus” (Cell, Volume 152, Issue 6, 2013), “Genome architecture and expression” (Current Opinion in Genetics & Development, Volume 23, Issue 2, 2013; Volume 25, April 2014). Here we mainly focus on the discoveries and evidence regarding the four-dimensional organization of the plant cell nuclei. The readers are also referred to the special journal issues (Molecular Plant, Volume 3, Number 4, 2010; Plant Physiology, Volume 158, Issue 1, 2012).

## NUCLEAR ENVELOPE

The nuclear envelope (NE) in eukaryotic cells surrounds the cell nucleus and is composed of a double membrane, nuclear pore complexes (NPCs), and the lamina (Hetzer et al., 2005). The double membrane surrounding the nucleoplasm is composed of two distinct membranes: the outer nuclear membrane (ONM) continuous with the endoplasmic reticulum (ER) and the inner nuclear membrane (INM) harboring a unique set of membrane proteins interacting with chromatin or the lamina (Hetzer and Wente, 2009). The ONM and INM are separated by the perinuclear space, called as the NE lumen, which acts as a repository of calcium as in ER (Erickson et al., 2006; Bootman et al., 2009). The two membranes are fused at NPC responsible for nucleocytoplasmic trafficking and gene regulation (Ptak et al., 2014; Tamura and Hara-Nishimura, 2014). In addition, they are also connected by the linker of nucleoskeleton and cytoskeleton (LINC) complexes comprising KASH (Klarsicht, ANC-1, and syne/nesprin homology) and SUN (Sad1 and UNC-84) proteins on the ONM and INM, respectively (Sosa et al., 2012; Zhou et al., 2012). Chromatin associated with the NE has been described as silent chromatin, which often interacts with the nuclear lamina, while active chromatin interacts with the nuclear pore proteins or resides at inner nucleus (Akhtar and Gasser, 2007; Kalverda et al., 2008). INM proteins interact with the lamina and/or chromatin in a tissue-specific manner. Many NE components are known to participate in mitotic progression, suggesting the key role of the NE in the disassembly and reformation of the nucleus during cell division (Kutay and Hetzer, 2008). In addition, in plants that lack centrosomes, NE serves as a microtubule (MT) organizing center (MTOC) during mitosis (Stoppin et al., 1994; Canaday et al., 2000).

## THE NUCLEAR LAMINA IN METAZOANS AND LAMIN-LIKE PROTEINS IN PLANTS

The nuclear lamina underlining the INM is an important structure, and is mainly composed of lamins and their interacting proteins for supporting the NE, attaching chromatin domains to the nuclear periphery, and localizing some proteins to NE in metazoan cells (Burke and Stewart, 2013). Lamins have also been shown to regulate numerous nuclear processes, including DNA replication, transcription, and chromatin organization (Dechat et al., 2010). Although, lamins preferentially interact with transcriptionally silent chromatin, some genes can be active or activated at the nuclear lamina (Kumaran et al., 2008). The lamina-associated chromatin domains (LADs) vary among different cell types, suggesting that LADs change spatiotemporally (Van Bortle and Corces, 2013). In addition to the direct interaction with chromatin, lamins participate in the regulation of transcription through interactions with histones, chromatin associated proteins, and transcription factors, such as lamin B receptor (LBR), heterochromatin protein 1 (HP1), barrier to autointegration factor (BAF), and octamer transcription factor 1 (OCT1; Wilson and Foisner, 2010). Mutations in lamins or the related proteins have been known to result in a group of phenotypically diverse genetic disorders known as laminopathies with symptoms ranging from muscular dystrophy and neuropathy to premature aging syndromes (Schreiber and Kennedy, 2013). The nuclear lamins fall into two separate classes: A-type and B-type, which are considered to be evolutionary precursors of the intermediate filament superfamily (Peter and Stick, 2012). The lamin A and lamin C are the main A-type lamins encoded by a single gene *LMNA*. The B-type lamins, lamin B1 is encoded by *LMNB1*, and B2, B3 are encoded by *LMNB2* in humans and other mammals (Goldman et al., 2002). *LMNA* gene is developmentally regulated and expressed primarily in differentiated cells, whereas, all vertebrate cells express at least one B-type lamins (Goldman et al., 2002). *Caenorhabditis elegans* has only one lamin gene (*lmn-1*), *Drosophila melanogaster* has two lamin genes (*Dm0* and *C*), and the mammals have three lamin genes, *LMNA*, *LMNB1,* and *LMNB2*, which encode for at least seven protein isoforms (Dittmer and Misteli, 2011; Lyakhovetsky and Gruenbaum, 2014).

The existence of a plant nuclear lamina is debatable as *lamin* homologues have not been identified in their genome databases (Fiserova and Goldberg, 2010). The nucleoskeletal structure of the plant nucleus was examined by biochemical analysis and transmission electron microscopy in the early 1990s (Moreno Díaz de la Espina et al., 1991; Li and Roux, 1992; Masuda et al., 1993, 1997). The isolated plant nuclear matrix “plamina” (plant lamina) seems basically similar to the metazoan nuclear lamina (Espina, 1996). Even though plant lamins are absent, field emission scanning electron microscopy (feSEM) of the plant nucleus revealed the presence of a plant lamina-like structure attached to the INM and NPCs, with a highly organized filamentous pattern (Evans et al., 2009). Interestingly, the human LBR expressed in a plant cell localizes to the INM (Graumann et al., 2007; Evans et al., 2009). This indicates that the NE targeting machinery is functionally conserved between the plant and animal species. Hence, identification of lamin like proteins in plants is one of the major interests for plant researchers.

Immunological methods have been used to identify a few insoluble proteins including NIF (nuclear intermediate filaments) group of proteins (Pérez-Munive et al., 2012; Ciska and Moreno Diaz de La Espina, 2013). The nuclear matrix constituent proteins (NMCPs) in plants exhibit many structural and biochemical similarities with lamins including the domain organization, subnuclear distribution and solubility (Ciska et al., 2013). So far NMCPs are considered as the best candidate proteins that could function as lamins in plants. NMCPs have a predicted tripartite structure with a head, coiled coil rod, and tail domains similar to that of lamins (Masuda et al., 1997). Additionally, NMCPs assemble and disassemble during mitosis in a manner similar to lamins (Masuda et al., 1999). NMCP1 was first described in 1993 in carrot, as a residual protein of the nuclear matrix with a pI value similar to that of lamins (Masuda et al., 1993). The *N*-terminal region, and an NLS-linked motif RYNLRR in the tail domain are responsible for the localization of NMCP1 to nuclear periphery (Masuda et al., 1993; Ciska and Moreno Diaz de La Espina, 2013; Kimura et al., 2014). NMCP family proteins have been characterized in several different species of plants, suggesting that NMCPs are well conserved in plants, but not in metazoans, yeast, or bacteria (Ciska et al., 2013; Wang et al., 2013). All NMCP1-like proteins reported so far share a long coiled-coil domain with a moderate amino acid sequence similarity, while the head and tail domains exhibit remarkable divergence (Kimura et al., 2010). A total of 97 NMCP proteins from 37 plant genomes have recently been classified into 2 clusters based on sequence similarity, structural analogy, and phylogenetic relationship: the NMCP1 and NMCP2 (Kimura et al., 2010; Ciska et al., 2013; Ciska and Moreno Diaz de La Espina, 2013).

*Arabidopsis thaliana* carries 4 NMCP genes (*LINC/CRWN 1–4*). LINC1 belongs to NMCP1 class, whereas, LINC2 and LINC3 are NMCP1-like proteins (or classified as NMCP3), and LINC4 is an NMCP2 protein (Dittmer et al., 2007; Kimura et al., 2010; Ciska et al., 2013). Mutations in LINC1, 2 and 4 have been shown to result in smaller nuclear size and altered nuclear morphology, and LINC1 playing a predominant role followed by LINC4 (Dittmer et al., 2007; Dittmer and Richards, 2008; Sakamoto and Takagi, 2013; Wang et al., 2013). Reduction in nuclear volume without a commensurate reduction in endoreduplication levels can lead to an increase in nuclear DNA density, therefore these proteins were later named as CRWN (CROWDED NUCLEI) when the name LINC (LITTLE NUCLEI) was often confused with the LINC complexes (Wang et al., 2013). Interestingly, some of the double (*LINC1/2, 1/3, 1/4, 2/4*) and triple (*LINC1/2/4, LINC1/3/4*) *LINC* mutants show whole-plant dwarfing morphology (Dittmer et al., 2007; Wang et al., 2013). In addition to the dramatic effects of LINCs on nuclear size and morphology, the maintenance of internal organization of the nucleus also requires LINC proteins. Chromocenter organization was found to be altered in *LINC1/LINC2* double mutant and *LINC4* single mutant by visualizing the spatial organization of chromocenters, and scoring for chromocenter numbers (Wang et al., 2013). A more interesting observation is that LINC1 appears to localize to the condensing chromatin during mitosis, while the other three LINCs, similar to lamins, are dispersed in the cytoplasm from metaphase to anaphase, indicating an extraordinary role of LINC1 in association with chromatin (Sakamoto and Takagi, 2013). This is similar to histone H1, which also localizes to the nuclear periphery and nucleoplasm in the nuclei isolated from suspension-cultured tobacco BY-2 cells synchronized in S/G2 phase, and associates with chromosomes during mitosis (Hotta et al., 2007; Nakayama et al., 2008). Therefore, it will be interesting to understand the potential relationship between LINC1 and histone H1. Recently, a plant-specific protein of unknown function (KAKU4) on INM was shown to modulate nuclear morphology and physically interact with LINC1 and LINC4 (Goto et al., 2014). Taking together, it seems that plant cells have evolved with a unique lamina-like structure composed of plant-specific proteins. In addition to identifying proteins that form the plant lamina, given the multiple roles of lamin in metazoans, it will be of great interest to study the potential roles of these proteins on nuclear organization and other processes, in addition to formation of the nuclear skeleton.

## THE LINKER OF NUCLEOSKELETON AND CYTOSKELETON COMPLEX

Lamins interact with many components transiently or stably and mediate a diverse range of functions (Wilson and Foisner, 2010). One group of these lamin-binding proteins are the SUN domain proteins located in the INM which together with KASH proteins in the ONM form the nuclear-envelope bridge, the LINC complex (Starr and Fridolfsson, 2010). SUN domain proteins contain a highly conserved SUN domain in their C-terminal fragment that is required for KASH protein binding, while the other regions are rather diverse (Rothballer and Kutay, 2013). Both budding and fission yeast have only one SUN domain protein, *C. elegans* and *D. melanogaster* have two, and mammals have at least five with SUN1 and SUN2 expressed in most of tissues and organs, whereas others are restricted to male germline (Hiraoka and Dernburg, 2009; Razafsky and Hodzic, 2009). KASH proteins are taIL-anchored membrane proteins with large cytoplasmic domains interacting with actin filaments, MTs, intermediate filaments, or centrosomes. The KASH domain at the C terminus is necessary and sufficient for the localization of KASH proteins to the ONM (Starr and Fridolfsson, 2010; Sosa et al., 2012). Both budding and fission yeast have two KASH proteins, three are found in *C. elegans*, two in *D. melanogaster*, and six in vertebrates: nesprin-1 to nesprin-4, KASH5, and LRMP (Mellad et al., 2011; Noegel and Neumann, 2011; Morimoto et al., 2012). The crystal structure of the human SUN2-KASH complex revealed that LINC complexes are formed by binding of three KASH peptides to the domain interfaces of the trimeric SUN proteins (Sosa et al., 2012). In addition to their roles in maintaining nuclear morphology and membrane structure, organization of the cytoskeleton, transmitting forces at the NE and in anchoring and moving of the nucleus, LINC complexes can also organize the genome and regulate signaling, cell division, and apoptosis (Rothballer and Kutay, 2013).

In *Saccharomyces cerevisiae*, telomeres are anchored to the nuclear periphery during interphase in different ways (Gartenberg, 2009; Mekhail and Moazed, 2010). The SUN domain protein Mps3 anchors and clusters the telomeres at the NE though interaction with chromatin silencing factor Sir4 and telomerase subunit Est1, respectively (Bupp et al., 2007; Schober et al., 2009; Horigome et al., 2011). While in *Schizosaccharomyces pombe*, during interphase, centromere clustering at the NE near the site of the spindle pole body is mediated by SUN domain protein Sad1 and the nucleoplasmic adaptor Csi1, and disruption of this anchor results in defects in chromosome segregation and mitotic progression (Hou et al., 2012). The DNA damage response (DDR) and DNA repair are critical for maintaining genomic stability in which the SUN domain proteins also play an important role. Persistent DNA double strand breaks (DSBs) are shuttled to the nuclear periphery and are retained by Mps3 and Ku70/Ku80 in yeast cells (Oza et al., 2009). It was also found that SUN1 and SUN2 were involved in DDR through their interaction with DNA-dependent protein kinase (DNAPK) complex in mouse cells (Lei et al., 2012). The embryonic fibroblast cells derived from null *Sun1^-/-^ Sun2^-/-^* mouse display increased DNA damage and decreased perinuclear heterochromatin (Lei et al., 2012).

From yeast to humans, chromosomes are always anchored to the NE by LINC complexes in the prophase of meiosis. In *S. cerevisiae*, telomeres are anchored to the NE by the SUN domain protein Mps3 (SUN domain) protein in association with Ndj1 (meiosis-specific nuclear adaptor protein) that contacts telomere, and Csm4 (atypical KASH domain) protein that interacts with the actin cytoskeleton. (Conrad et al., 2007, 2008). In *S. pombe*, this is achieved by Sad1 (SUN domain) protein, Bqt1 and Bqt2 (meiosis-specific nuclear adaptor proteins) connecting Sad1 to telomeres, and Kms1 (KASH domain) protein through interactions with MTs and dynein on the cytoplasmic side. (Miki et al., 2002; Chikashige et al., 2006). In mammals, tethering of meiotic chromosomes to the NE requires SUN1 and KASH5 which colocalize with dynein; however, the adaptor proteins that connect SUN1 to telomeres are still unknown (Ding et al., 2007; Koszul et al., 2008; Morimoto et al., 2012; Rothballer and Kutay, 2013). In mitosis, SUN1 and SUN2 facilitate the removal of NE/ER membranes from chromatin, in early prometaphase when the NE breaks down. Consistent with this observation, depletion of SUN1/2 affects spindle assembly and cell cycle progression (Turgay et al., 2014).

Although a very few animal INM proteins have homologs in plants, two divergent classes of SUN proteins exist in plants (Murphy et al., 2010). Two SUN proteins, AtSUN1 and AtSUN2 from *Arabidopsis* were shown to interact with three WPP domain-interacting proteins (WIPs): AtWIP1, AtWIP2, and AtWIP3, which share a low degree of similarity with metazoan KASH proteins (Graumann et al., 2010; Oda and Fukuda, 2011; Zhou et al., 2012; Zhou and Meier, 2013). AtSUN1 and AtSUN2 have structural similarities with the SUN-domain proteins identified in other species such as an *N*-terminal domain with a NLS, a transmembrane domain, a coiled-coil domain, and a highly conserved C-terminal SUN domain (Graumann et al., 2010; Zhou et al., 2012). AtSUN1 and AtSUN2 form homomers and heteromers through their coiled-coil domains and are localized to the NE with low mobility through their *N*-termini and coiled-coil domains (Graumann et al., 2010; Oda and Fukuda, 2011). AtWIPs are plant specific ONM proteins which function to anchor the RanGTPase-activating protein 1 (AtRanGAP1) to the NE (Xu et al., 2007a). AtSUNs interact with AtWIP1 through their SUN domains and are required for targeting AtWIP1 and AtRanGAP1 to the NE (Zhou et al., 2012). AtSUN1 also affects the mobility of AtWIP1 and AtLINC1/CRWN1-YFP (Zhou et al., 2012; Graumann, 2014). Similar to AtLINCs/CRWNs and the nucleoporin AtNup136, AtWIPs and AtSUNs are necessary to maintain the elongated nuclear shape (Dittmer et al., 2007; Tamura et al., 2010; Zhou et al., 2012).

## THE CHANNEL AND BEYOND THE CHANNEL BETWEEN NUCLEUS AND CYTOPLASM: THE NUCLEAR PORE COMPLEX

Another major component of the NE is the NPCs, anchored in the lamina and studded throughout NE at the ONM and the INM fusion sites. NPCs mediate selective transport of molecules between the nucleus and cytoplasm (Wente and Rout, 2010). NPCs consist of multiple copies of ∼30 different proteins known as nucleoporins (or nups), which form an evolutionarily conserved eightfold-symmetrical structure comprising of a NE-embedded scaffold that surrounds a central transport channel and the cytoplasmic and nuclear rings attached with eight filaments loosing outside or joined the nuclear basket, respectively (D’Angelo and Hetzer, 2008). The core scaffold of the NPC, which includes Nup84-subcomplex and Nup170-subcomplex, lines the circumference of the pore where it interacts with the pore membrane and membrane proteins, and supports the Nups rich in phenylalanine-glycine (FG) residue repeats that occupy the central channel and play a central role in transport (Ptak et al., 2014). A growing body of experimental evidence suggests that the FG-Nups around the central channel and other Nups that form the nuclear basket also play important roles in modulating chromatin structure and gene expression (Strambio-De-Castillia et al., 2010).

Functional compartmentalization and regulation of the genome depend on the interactions between genomic regions and various nuclear scaffolds and macro-molecular complexes, such as the lamina and NPCs (Pascual-Garcia and Capelson, 2014). As was mentioned earlier, heterochromatin tends to concentrate at the nuclear periphery; however, the peripheral heterochromatic landscape is disrupted near NPCs (Ptak et al., 2014). In contrast to the lamina, the NPC has been recognized as a transcriptionally permissive region for many inducible genes (Meldi and Brickner, 2011). These active genes interact with Nups present in different parts of NPC and in nucleoplasm. Therefore, Nups also serve as chromatin-associated factors, and play roles in transcriptional activation or repression, transcript elongation and processing, transcriptional memory, coupling of transcription and mRNA export, and genome integrity (Arib and Akhtar, 2011; Ptak et al., 2014).

The structure and organization of plant NPCs closely resemble that of the known yeast and vertebrate NPCs (Roberts and Northcote, 1970; Fiserova et al., 2009). Although several proteins with significant similarity to animal and yeast Nups have been identified by genetic approaches, it is difficult to identify Nup proteins in plants solely based on sequence similarity (Boruc et al., 2012). Until recently only a few nucleoporins were identified and characterized in plants. In 2010, an interactive proteomic approach by immunoprecipitation coupled with mass spectroscopy using a GFP fused nucleoporin, GFP-RAE1 (RNA export factor 1), was used to identify *Arabidopsis* nucleoporins (Tamura et al., 2010). This approach has identified and characterized 8 known and 22 novel Nups including Nup136/Nup1, a unique nucleoporin with no vertebrate homolog (Tamura et al., 2010). The homologs of human Nup358, Nup188, Nup153, Nup45, Nup37, NUCLEAR DIVISION CYCLE1 (NDC1), and pore membrane protein121 (Pom121) were not identified in this experiment (Tamura et al., 2010). However, earlier genomic data indicated that NDC1 is evolutionary conserved and AtNDC1 was predicted to contain six transmembrane domains shared by all NDC1 proteins (Mans et al., 2004; Stavru et al., 2006).

Nups in plants affect many processes including nuclear morphogenesis, pollen development, regulation of flowering-time, overall plant development, plant–microbe interactions, hormone signaling, and cold-stress tolerance (Xu and Meier, 2008). The unique plant Nup136, which exhibits a more dynamic behavior on the NE than other Nups, is considered as a functional homolog to animal Nup153, although they have no sequence homology (Tamura et al., 2010). The mutant of *nup136* has more spherical and uniform nuclei, whereas overexpression of Nup136-GFP was found to induce elongation of nuclei in various tissues, including the guard cells, rosette leaf epidermal cells, trichome cells (Tamura et al., 2010; Tamura and Hara-Nishimura, 2011). It will be necessary to determine whether the effect of Nup136 on nuclear shape is related to altered endoreduplication or interactions with other factors responsible for the maintenance of nuclear morphology, such as AtSUNs, AtWIPs, and AtLINCs. Furthermore, the *nup136* mutant also showed developmental defects, with stunted fruits, substantially fewer mature seed grains and early flowering (Tamura et al., 2010). Strikingly, similar phenotypes, including dwarfism, early flowering and/or other developmental defects, such as infertility and abnormal meristem resulting in spiral phyllotaxy were seen among some *Nup* mutants, including *nup96* (Parry et al., 2006), *nup160* (Dong et al., 2006), *tpr/nua* (Jacob et al., 2007; Xu et al., 2007b), and the overexpression-based co-suppression line of *AtNup62* (Zhao and Meier, 2011). These pleiotropic phenotypes indicate that the NPCs play a critical role in plant development (Li et al., 2008; Merkle, 2011; Parry, 2013).

Apart from the above-mentioned developmental roles, Nups also regulate specific pathways. Nup160, which is required for plant tolerance to cold stress, together with Nup96, were identified as the suppressor of auxin resistance1 and 3 (sar1 and sar3) respectively, in a screen for suppressors of the auxin-resistant *Arabidopsis* mutant *axr1*, probably due to their influence on the localization of transcriptional repressor AXR3/IAA17 and/or mRNA export (Dong et al., 2006; Parry et al., 2006; Robles et al., 2012). AtTpr was also shown to have a similar role in the suppression of auxin-resistant phenotype of *axr1* (Jacob et al., 2007). Nups have also been shown to be involved in the plant-pathogen interaction. In a screen aimed to identify suppressors of *snc1* (*suppressor of npr1-1, constitutive 1*) mutants with constitutive resistance to a range of fungal and bacterial pathogens, a range of *mos* (*modifier of snc1*) mutants that rescue a majority of *snc1* phenotypes were identified, and most of MOS proteins including two Nups, MOS3/NUP96, and MOS7/NUP88 were predicted to play a role either in RNA processing or nuclear transport (Zhang, 2005; Cheng et al., 2009). A reverse genetics approach was used to examine a potential role of additional subunits of the predicted *Arabidopsis* Nup107–160 complex in plant immunity. Two members of the NUP107–160 complex, nup160 and seh1, were found to contribute to pathogen defense (Roth and Wiermer, 2012; Wiermer et al., 2012). In *Mos7-1* mutant, the nuclear accumulations of SNC1, enhanced disease susceptibility 1 (EDS1) and non-expresser of PR genes 1 (NPR1) involved in defense signaling were significantly reduced, suggesting that MOS7/NUP88 regulates plant defense by specifically modulating the nuclear concentrations of certain defense proteins (Cheng et al., 2009). In contrast, NUP160 and SEH1 probably work through regulating nuclear mRNA export and EDS1 protein accumulation (Roth and Wiermer, 2012; Wiermer et al., 2012). In *Lonicera japonicas*, Nup85 and Nup133 were found to affect processes including Ca^2+^ spiking, rhizobial and fungal symbiosis, and seed production (Kanamori et al., 2006; Binder and Parniske, 2014). *Nup85* and *Nup133* mutants exhibit severe defects in the temperature dependent growth and development (Kanamori et al., 2006; Binder and Parniske, 2014).

In summary, nuclear pores are involved in many pathways from developmental regulation to stress signaling. However, the molecular mechanisms were only roughly explained by the transport of various factors, including RNAs and proteins essential for corresponding processes (Tamura and Hara-Nishimura, 2014). Therefore, the transport-independent functions of plant Nups, like Nup-chromatin interactions in spatio-temporal organization of gene expression remains to be explored (Boruc et al., 2012).

## THREE-DIMENSIONAL ORGANIZATION OF THE GENOME

The genome is non-randomly organized in the three-dimensional (3D) nuclear space of cell nucleus to facilitate its appropriate expression (Gibcus and Dekker, 2013). High-order genome organization is cell-type specific and is modulated by cellular processes, such as proliferation, differentiation, and stress. Hence, unraveling the mechanism and regulation of genome organization is critical to understand the genome functions (Meaburn and Misteli, 2008; Schwartz and Hakim, 2014). The study of 3D genomics with a focus on the 3D structure and function of the whole genome has become an important part of the current genomics era. Recent findings using the ChIA-PET (Chromatin Interaction Analysis by Paired-end Tag Sequencing) and Hi-C showed the influence of the 3D genomic structure on genome functions (Li et al., 2014). Transcriptional control and other processes including DNA repair, DNA replication, and X chromosome inactivation have been shown to be influenced by 3D genome organization (Gibcus and Dekker, 2013; Gorkin et al., 2014). A hierarchy of structures is involved in the proper folding of each of the large complex chromosomes, ranging from chromatin loops that connect genes and enhancers to large chromosomal domains and nuclear compartments (Gibcus and Dekker, 2013). One of the basic levels of eukaryotic genomic organization is the chromosome territory (CT) where each chromosome occupies a distinct sub-nuclear volume. CT is common to yeast, plants, and animals (Cremer and Cremer, 2010; Schwartz and Hakim, 2014). The existence of CT was demonstrated by fluorescence *in situ* hybridization (FISH) technology using probe sets designed to paint entire chromosomes (Bolzer et al., 2005). CTs are spatially distinct with considerable intermingling between different chromosomes near the borders of CTs (Branco and Pombo, 2006; Gorkin et al., 2014). Smaller chromosomes are generally located in the interior and larger chromosomes toward the periphery of the nucleus (Sun et al., 2000). Besides the size of chromosome, gene density can also influence the location of CT. For example, the gene-rich human chromosome 19 is located in a more central position in the nucleus, whereas, the similarly sized, but gene-poor chromosome 18 was found at the nuclear periphery (Croft et al., 1999; Boyle et al., 2001; Cremer and Cremer, 2001). Within a CT, the position of specific regions are non-random and often correlate relative to their transcriptional activity (Gorkin et al., 2014). Gene-rich regions prefer to localize to the periphery of CTs, and specific regions can shift from the interior to the periphery based on their activities during development (Chambeyron and Bickmore, 2004; Morey et al., 2007; Boyle et al., 2011). However, the details on the relationship between CT positioning and transcriptional activity remain unclear, and shifts in CT position do not always alter the transcriptional activity (Gorkin et al., 2014). In addition to the organization of CTs, it was suggested that chromosomes are organized into topologically associating domains (TADs), the building blocks of CTs, which are relatively invariant across cell types and conserved between mouse and human (Dixon et al., 2012; Schwartz and Hakim, 2014). As intra-chromosomal contacts are more frequent than inter-chromosomal ones, TADs located within the chromosome associate more frequently than those located on different chromosomes. In addition, chromosomal loci within the TADs associate more frequently than those between TADs (Gibcus and Dekker, 2013; Tanay and Cavalli, 2013; Schwartz and Hakim, 2014). The enhancers and other regulatory elements often communicate with each other and with the promoters of genes within the same TAD. For example, Hox genes are regulated by several different distal enhancers within the same TAD (Montavon et al., 2011; Noordermeer et al., 2011). Binding sites of the protein CTCF, which functions as a transcriptional insulator, are highly enriched at TAD boundaries. Deletion of a TAD boundary containing CTCF binding sites increases the interactions between adjacent TADs (Dixon et al., 2012; Nora et al., 2012; Gorkin et al., 2014). Genomic regions with similar transcriptional activity normally associate with each other, as the transcription-active regions frequently interact in space with other active loci and regions that lack transcriptional activity tend to interact with other inactive regions as demonstrated by Hi-C technologies (Simonis et al., 2006; Lieberman-Aiden et al., 2009; Gorkin et al., 2014). These distinct active and inactive interaction networks are referred to as A and B compartments respectively, reflecting the segregation of euchromatin and heterochromatin in space (Gorkin et al., 2014). Moreover, inactive chromatin tends to associate primarily with inactive nuclear landmarks, such as the nuclear lamina and nucleolar periphery, while the position and translocation of active chromatin are more difficult to characterize (Schwartz and Hakim, 2014). Similar to the rRNA gene clusters from different chromosomes colocalize at the nucleolus for the transcription by RNA polymerase I, genes transcribed by RNA polymerase II have also been shown to colocalize together at transcription factors (Edelman and Fraser, 2012; Papantonis and Cook, 2013; Gorkin et al., 2014). Although TAD domains are essentially invariant across different cell types, the connectivity of the intra-domain and inter-domain is cell type specific and is dictated by lineage specific transcription factors (Schwartz and Hakim, 2014). It is suggested that cell type specific transcription factors associated with the genome play a predominant role in configuring the higher order genome organization. However, the role of transcription factors as nuclear organizers and the linkers between transcriptional activity and co-localization of genomic loci remain controversial (Hakim et al., 2011; Rieder et al., 2014; Schwartz and Hakim, 2014). When DNA lesions occur at random locations, the probabilities of chromosomal translocations correlate well with the frequency of chromosomal contacts. The targeted DNA damage at specific chromosomal loci, such as programmed DNA damage that occur during B cell antibody diversity in B cells, is a major contributor to the observed translocation frequency at its interacting chromosomal loci (Schwartz and Hakim, 2014).

These conclusions are largely addressed or confirmed by the revolutionary Chromosome Conformation Capture (3C) technology and its derivatives, together with automation and computation of image acquisition and analysis. These studies have provided unprecedented genome-wide perspectives on the spatial relationships of DNA sequences, both within and between chromosomes (Dostie and Bickmore, 2012). As the 3C technology was successfully used both in animal-model systems and yeast, plant-specific 3C protocols, particularly for maize, were documented (Dekker et al., 2002; Louwers et al., 2009; Hovel et al., 2012). Recently, circular chromosome conformation capture (4C) technology was used to characterize the chromosomal architecture of *Arabidopsis* (Grob et al., 2013). *Arabidopsis* genome seems to be packed in a predictive manner with heterochromatin and euchromatin representing two distinct interactomes. Chromosome interactions relate with the linear position on the chromosome arm, for example, distal chromosome regions have more potential to interact with other chromosomes (Grob et al., 2013). A gene loop containing the floral repressor FLC was identified using 3C and was found to be disrupted during vernalization (Crevillen et al., 2013). It is predictable that large amounts of data in this field will emerge in the coming years. It is anticipated that the 3D-genome organization field will continue to produce large data sets, and therefore, it is important to develop improved statistical and computational tools (Dekker et al., 2013a).

Most studies on chromosome organization and dynamics in plants in the past few years are based on FISH and live-imaging techniques (Tiang et al., 2011). CTs were first visualized in *Arabidopsis* using FISH in 2001 (Lysak et al., 2001). The CT arrangement and homologous pairing were found to be predominantly random except for chromosomes 2 and 4, which bear nuclear organizer regions (NORs) in *Arabidopsis* (Pecinka et al., 2004). NOR-bearing chromosomes also tend to associate with each other, likely because of their association with the nucleolus (Pecinka et al., 2004; Berr and Schubert, 2007). Chromosome arrangement and nuclear architecture are conserved between *Arabidopsis thaliana* and *A. lyrata,* although the centromeric sequences are different (Berr et al., 2006). Chromosomes in many plant species adopt Rabl configuration during interphase when the centromeres and telomeres are located at opposite sides of the nucleus. However, *Arabidopsis* exhibit a strikingly different type of chromatin arrangement with telomeres clustering around the nucleolus and centromeres positioning at the nuclear periphery (Armstrong et al., 2001; Cowan et al., 2001; Fransz et al., 2002; Fang and Spector, 2005). The rosette-like structure of *Arabidopsis* CTs is formed from the euchromatic loops of 0.2 to 2 Mb from the chromcenters, the distinct dense bodies of centromeric heterochromatin, which contains the majority of genomic repeats and exhibits epigenetic marks of inactive chromatin (Fransz et al., 2002; Tiang et al., 2011). The organization of endoreduplicated sister centromeres is cell type dependent, clustering in root epidermal cells and dispersed in leaf epidermal cells (Fang and Spector, 2005). Endoreduplication-driven polyploidy has been found to affect chromosome arrangement by reducing the speed and increasing the freedom of chromosome movement (Kato and Lam, 2003; Berr and Schubert, 2007). In *AtLINC/AtCRWN* mutants, altered chromosome arrangement, slightly changed endoreduplication, and altered nuclear size and shape were observed, but we still have no idea about which is the cause or result (Dittmer et al., 2007; Sakamoto and Takagi, 2013; Wang et al., 2013). Light-regulated large-scale reorganization of chromatin was reported during the floral transition with chromocenters being smaller before the transition and restored after (Tessadori et al., 2007). A study on the nuclear phenotypes of *Arabidopsis* from different geographical origins and habitats has suggested that natural variation in chromatin compaction is likely to be dependent on light intensity, demonstrating a positive correlation with latitude of geographic origin, with PHYTOCHROME-B (PHYB) and HISTONE DEACETYLASE-6 (HDA6) as positive regulators (Tessadori et al., 2009).

Chromosomes in mitosis are more dynamic compared to their limited movements in interphase. During the prophase to metaphase transition, condensed chromosomes tend to relocate to the center of the cell while their centromeres rotate gradually to orient perpendicular to the metaphase plate. In anaphase, centromeres direct the chromosomes towards the opposite poles (Fang and Spector, 2005; Tiang et al., 2011). Following anaphase, centromere arrangements in two daughter cell nuclei are clearly asymmetrical and show significant differences in their 3-D distribution compared to the mother cell nucleus, although the centromeres in both mother and daughter cell nuclei locate at the nuclear periphery (Fang and Spector, 2005). This is consistent with a study in HeLa cells, which showed large-scale CT arrangements change from one cell cycle to the next (Walter et al., 2003). However, it was also reported that chromosome positions often exhibit mirror symmetry in daughter cells immediately after mitosis (Gerlich et al., 2003; Berr and Schubert, 2007). In plants, dynamic chromosomes in meiotic prophase I undergo major reorganization, such as chromosome condensation, establishment of meiotic-specific chromosome structure, homologous chromosome pairing, and dynamic chromosome movements (Tiang et al., 2011). In early meiosis, adoption of the meiosis-specific chromosome structure by chromatin condensation in leptotene stage is one of the key processes in meiotic prophase I (Dawe et al., 1994; Golubovskaya et al., 2006). Incidently, the transcriptome analyses of *Arabidopsis* meiocytes showed that a staggering number of genes are expressed during the meiotic prophase I (Chen et al., 2010). The following homologous chromosome pairing was shown to be tightly linked to the progression of meiotic recombination (Tiang et al., 2011). For a more detailed description of the chromatin in meiosis, the readers are referred to the reviews (Pawlowski, 2010; Ronceret and Pawlowski, 2010; Tiang et al., 2011).

## NUCLEAR BODIES

In the dynamically organized cell nucleus, CTs harbor a variety of functionally distinct nuclear bodies (NBs). NBs are highly dynamic structures, enriched with proteins and/or RNAs involved in similar processes in a constrained space, presumably to improve reaction efficiency and/or regulation (Sleeman and Trinkle-Mulcahy, 2014). In addition to serving as reaction sites to efficiently facilitate specific biological processes, NBs can also act as hubs for regulating the expression of recruited gene loci or as storage/modification sites for recycling and modifying RNA or protein molecules (Mao et al., 2011b). Recently, in a human whole genome wide screen using markers of known NBs, 325 proteins were identified to localize to distinct NBs, including nucleoli, promyelocytic leukemia nuclear bodies (PMLNBs), nuclear speckles, paraspeckles, cajal bodies (CBs), Sam68 NBs, polycomb bodies, and other uncharacterized NBs (Fong et al., 2013). In addition, biochemical approaches have been used to identify the protein components in nucleoli and speckles/interchromatin granule clusters (Andersen et al., 2002; Scherl et al., 2002; Saitoh et al., 2004). Using a modified RNA tagging and recovery of associated proteins (TRAP) method, known as Immuno-TRAP, gene loci associated with PML NBs were recently identified (Ching et al., 2013).

All these approaches were aimed to reveal the novel components of the NBs; the question is how these individual components assemble and interact with each (Matera et al., 2009). Three models have been proposed to explain the assembly of NBs through dynamic interactions between the individual components: (1) stochastic assembly model, in which each component contributes equally, and the assembly process is largely random; (2) ordered assembly model, in which hierarchically individual components follow a tightly controlled sequential order to assemble one after another; (3) seeding assembly model, in which RNAs or proteins hierarchically different from the other components serve as seeds to initiate and nucleate the formation of a nuclear body, subsequently followed by either stochastic or ordered assembly (Dundr and Misteli, 2010; Mao et al., 2011a,b). Using a bacterial Lac operator/repressor (LacO/LacI) tethering system, CB was found to be *de novo* assembled via stochastic interactions (Kaiser et al., 2008). In contrast, nucleoli were found to be assembled through an “RNA-seeded” model, likely triggered by activation of rDNA transcription (Karpen et al., 1988). Nucleations of histone locus bodies, speckles, paraspeckles, and nuclear stress bodies have been shown to follow “RNA-seeded” model (Mao et al., 2011a; Shevtsov and Dundr, 2011). Similar to LacO/LacI tethering system used in the assembly of nuclear body, LacI-Lamin B fusion protein was developed to tether LacO locus to the nuclear lamina in mammalian cells (Kumaran and Spector, 2008; Dundr, 2013). Recently, nucleolus-tethering system (NoTS) was developed to tether a protein to nucleolus in plant cells (Liu et al., 2014). These tethering systems have greatly enabled to test models of nuclear organization.

Plant NBs include the nucleolus, CBs, nuclear speckles, cyclophilin-containing speckles, nuclear dicing bodies (or D-bodies), photobodies, and AKIP1-containing NBs. Nucleolus and CBs were characterized in details as early as in 1990s (Beven et al., 1995, 1996; Boudonck et al., 1998, 1999; Brown and Shaw, 1998; Shaw et al., 1998; Acevedo et al., 2002; Cui and Moreno Diaz de la Espina, 2003). For detailed information on nucleolus, recent reviews are recommended (Shaw and Brown, 2012; Stepinski, 2014). CBs are likely to be involved in the maturation and transport of snRNPs and snoRNPs (Sleeman et al., 2001; Gall, 2003). In *Arabidopsis*, the number of CBs per nucleus is regulated by cell type, developmental stage, and cell cycle parameters in the root epidermis and during pollen development and pollen tube growth (Boudonck et al., 1998; Scarpin et al., 2013). Using a green fluorescent protein (GFP) fusion to the spliceosomal protein U2B”, CBs were shown to be very dynamic, moving within the nucleus into the nucleolus, and fusing together (Boudonck et al., 1999). In *Arabidopsis*, a screen for mutants with altered CBs has identified *ncb1* (no cajal bodies 1) with no CBs due to a single base change at a splice site in Atcoilin, a distant homolog of vertebrate coilin (Collier et al., 2006). Atcoilin is required for cajal body formation, though *Arabidopsis* plants lacking CBs are viable and appear normal (Collier et al., 2006). It was reported that CBs and the nucleolus are required for the systemic infection of plants by viruses and atcoilin was found to be involved in the interactions between plants and viruses (Shaw et al., 2014). ARGONAUTE4 (AGO4), the small RNA-binding protein involved in RNA-directed DNA methylation (RdDM), was found to be localized to CBs and other sites (Li et al., 2006, 2008). Nuclear speckles, considered as storage sites for splicing factors, are often observed near active transcription sites with pre-mRNAs in fibrillar structures outside the speckles (Fang et al., 2004; Spector and Lamond, 2011). Speckles are dynamic structures with changes in size, shape, and number in response to temperature, stress, and status of transcription or phosphorylation (Reddy et al., 2012). CypRS64 and CypRS92, two RS domain containing cyclophilins, have been identified to interact with SR proteins and localize to a small number of novel NBs called cyclophilin-containing speckles, and are distinct from CBs (Lorkoviæ et al., 2004). AKIP1 is a RNA-binding motifs containing protein with homology to heterogeneous nuclear RNA (hnRNP)-binding protein A/B, and was reported to relocate into speckle-like domains when treated with abscisic acid (Li et al., 2002). Dicing bodies, which contain microRNA-processing proteins like DICER-LIKE1 (DCL1) and HYPONASTIC LEAVES1 (HYL1) were found to be essential for the accurate processing of primary microRNAs (Fang and Spector, 2007; Song et al., 2007; Liu et al., 2013).

An amazing example of dynamic nuclear organization in plants is the light-regulated photobodies. Phytochromes (phys) include the red (R) and far-red (FR) light receptors, which can interconvert between two relatively stable conformers: Pr and Pfr (Rockwell et al., 2006). Phys rapidly translocate from the cytoplasm to the nucleus upon photoactivation of Pr to the Pfr. During this process the photobodies containing both phyA and phyB can be observed after exposure to light for 1–2 min (Chen, 2008). PhyA mediates responses during the dark-to-light transition, accumulates in the dark, and rapidly degrades under R light, forming transient/early photobodies (Chen, 2008). PhyB on the other hand is the major phytochrome, which mediates responses under R light and is relatively stable, forming stable/late photobodies (Chen, 2008). The size and number of phyB-containing photobodies under continuous R light is determined by the percentage of phyB in the Pfr; under low intense R light, low R-to-FR ratio, phyB were observed in many smaller photobodies or diffused in the nucleoplasm. Under high-intense R light, high R-to-FR ratio, phyB exclusively localized to a few large photobodies were observed (Chen et al., 2003; Chen, 2008; Van Buskirk et al., 2012). The changes tightly correlate with the light-dependent hypocotyl inhibition. In addition to the photoreceptors, COP1 (CONSTITUTIVELY PHOTOMORPHOGENIC 1) and several other light-regulated proteins including transcription factors also form photobodies. The assembly of NBs was recently revealed by a NoTS (Liu et al., 2014). In this system, different components involved in the light signaling pathways were tethered to the nucleolus by fusing them with nucleolin2 (Nuc2), a nucleolar marker protein, for analyzing the initiation of photobodies. The assembly of photobodies was evaluated by visualizing the fused protein in body-like structures containing other components at the periphery of the nucleolus (Liu et al., 2014). COP1, phyB, cry1, cry2, and PIF7 fusion proteins all formed body-like foci at the periphery of the nucleolus containing other components in photobodies. Interestingly, COP1, cry1, cry2, UVR8, and CO were found in Nuc2-COP1 bodies, COP1 in Nuc2-cry1 and Nuc2-cry2 bodies, PIF7 in the Nuc2-phyB bodies, and phyB in Nuc2-PIF7 containing bodies, indicating that the assembly of photobodies follows a self-organization model (Liu et al., 2014).

## CONCLUDING REMARKS

Taking advantage of the recent developments in imaging and 3C technologies, we now have a better understanding of the eukaryotic cell nucleus as a highly ordered structure, harboring organized chromatin territories and various NBs in spatial-temporal dynamics. The transcriptional activity of chromatin is tightly regulated through interaction with its self and other major nuclear compartments, such as the lamina and NPCs. Many NBs which can serve as storage/modification or reaction sites contribute to gene regulation, post-transcriptional processing and/or modification. The nuclear organization of the animal cell is the best studied, with studies on plants relatively lagging behind. Therefore, it will be of great general interest to further understand the four-dimensional organization and function of the plant sub-nuclear compartments.

## Conflict of Interest Statement

The authors declare that the research was conducted in the absence of any commercial or financial relationships that could be construed as a potential conflict of interest.
